# Naval

**Published:** 1870-11

**Authors:** 


NAVAL.—*Dr. Heman P. Babcock and Naval Rank.*

We are happy to see that our friend Dr. Babcock has settled down at Oakland, California. Dr. Babcock is a native of Buffalo, and a graduate of our Medical College. Shortly before the war for the Union, he entered the navy as assistant surgeon, and during the whole of the struggle his professional services were in constant requisition, and he always acquitted himself with great credit. Some two years since he resigned from the navy, and engaged as surgeon in the more pleasant and lucrative service of the Pacific Mail Steam-ship Company. He has several times made the transit between San Francisco and Japan, and now, after a large and varied experience, has very wisely determined to devote himself to private practice. Oakland is a pleasant and beautiful suburb of San Francisco, lying just across the bay, and we hope that Dr. Babcock will find it both a pleasant and profitable residence. We commend him to our *confreres* in California, as a very intelligent, energetic and honorable physician, and we shall hope to publish occasionally contributions from his ready and facile pen. Below we publish resolutions introduced by Dr. Babcock, at a meeting of the Alameda Connty Medical Association, held at its Rooms, in Oakland, October 17th, 1870, which were unamiously dopted:

WHEREAS, Of late, repeated and persistent insults have been offered our professional brethren in the U. S. Navy. by the authority of the Navy Department, degrading them in rank and position; lessening by example the respect due their profession, and contracting their sphere of usefulness; and

WHEREAS, In every civilized community throughout the world, save in our Navy, the profession of medicine is considered, at least, equal in dignity and respectability to any other profession; and

WHEREAS, In our service, the members of the medical staff are selected by competitive examination from among the graduates of our medical schools, while the line officers are selected to be educated at our country's expense from among the uneducated boys of the community, by favoritism, by relationship, or, as has lately been proven, by purchase; and

WHEREAS, Rank and command are distinct ideas, having no necessary connection; there being a recognized necessity for *one* commander in all military operations, to show to whom the other officers are subordinate for the time being; and

WHEREAS, If physical courage and personal exposure are the only tests of merit, no corps can show, during the late war for example, a larger proportion of killed by the enemy, by fire, by water, or by the more deadly and insiduous foe—disease, than the medical officers of the Navy; therefore be it

*Resolved*, That we consider the stigma to which they have been subjected as applying to the profession at large, and, while it is unremoved, we consider that no young medical man, having a proper regard to his self respect, can accept an appointment in the medical corps of the Navy, and subject himself and his profession to the indignities which the self-constituted and newly-born "Aristocracy of the Line" impose.

*Resolved*, That we view with pain and sympathy the position of the senior officers of the medical corps, whose long service now renders it impossible for them to resign and commence life anew: and we call upon our Senators and Representatives in Congress to recognize their position as co-equal with the highest in the service, by giving them military rank, such as is justly enjoyed by the medical staff of the Army, and by that in the services of each of the civilized nations of the world, together with such increased emoluments and promotions as will recognize their invaluable services to our country, and recompense them for the insults and oppression to which they have most unjustly been subjected.

*Resolved*, That a copy of these resolutions be sent to each Senator and Representative from this State, and that our delegates to the State Medical Society be instructed to bring this subject before that body for its action.

(Signed)

(Signed) T. H. PINKERTON, M. D., President

S. C. HOLMES, M. D, Secretary.

